# Steering Bell-diagonal states

**DOI:** 10.1038/srep22025

**Published:** 2016-02-25

**Authors:** Quan Quan, Huangjun Zhu, Si-Yuan Liu, Shao-Ming Fei, Heng Fan, Wen-Li Yang

**Affiliations:** 1 Institute of Modern Physics, Northwest University, Xi’an 710069, China; 2 Institute for Theoretical Physics, University of Cologne, Cologne 50937, Germany; 3 Perimeter Institute for Theoretical Physics, Waterloo, Ontario N2L 2Y5, Canada; 4 School of Mathematical Sciences, Capital Normal University, Beijing 100048, China; 5 Max-Planck-Institute for Mathematics in the Sciences, Leipzig 04103, Germany; 6 Institute of Physics, Chinese Academy of Sciences, Beijing 100190, China; 7 Collaborative Innovation Center of Quantum Matter, Beijing 100190, China; 8 Center for Mathematics and Information Interdisciplinary Sciences, Beijing, 100048, China

## Abstract

We investigate the steerability of two-qubit Bell-diagonal states under projective measurements by the steering party. In the simplest nontrivial scenario of two projective measurements, we solve this problem completely by virtue of the connection between the steering problem and the joint-measurement problem. A necessary and sufficient criterion is derived together with a simple geometrical interpretation. Our study shows that a Bell-diagonal state is steerable by two projective measurements iff it violates the Clauser-Horne-Shimony-Holt (CHSH) inequality, in sharp contrast with the strict hierarchy expected between steering and Bell nonlocality. We also introduce a steering measure and clarify its connections with concurrence and the volume of the steering ellipsoid. In particular, we determine the maximal concurrence and ellipsoid volume of Bell-diagonal states that are not steerable by two projective measurements. Finally, we explore the steerability of Bell-diagonal states under three projective measurements. A simple sufficient criterion is derived, which can detect the steerability of many states that are not steerable by two projective measurements. Our study offers valuable insight on steering of Bell-diagonal states as well as the connections between entanglement, steering, and Bell nonlocality.

Einstein-Podolsky-Rosen (EPR) steering^1^, as noticed by Schrödinger^2^, is an intermediate type of nonlocal correlations between entanglement and Bell nonlocality. In the framework of modern quantum information theory, this “spooky action” can be described as a task of entanglement verification with an untrusted party, as explained by Wiseman *et al.*^3^,^4^. It hinges on the question of whether Alice can convince Bob that they share an entangled state, despite the fact that Bob does not trust Alice. In order to achieve this task, Alice needs to change Bob’s state remotely in a way that would be impossible if they shared classical correlations only. Contrary to entanglement and Bell nonlocality, steering features a fundamental asymmetry because the two observers play different roles in the steering test^3^,^4^,^5^. Recently, growing attention has been directed to steering because of its potential applications in quantum information processing, such as quantum key distribution (QKD)^6^, secure teleportation^7^, and entanglement assisted subchannel discrimination^8^.

Two basic questions concerning steering are its detection and quantification. One approach for detecting steering is to prove the impossibility of constructing any non-steering model^3^,^4^. A practical alternative is to demonstrate the violations of various steering inequalities^9^,^10^,^11^,^12^,^13^,^14^. The first steering inequality was derived by Reid in 1989 9, which is applicable to continuous variable systems, as considered in EPR’s original argument. General theory of experimental steering criteria were developed in ref. 10, followed by many other works^11^,^12^,^13^,^14^. In line with theoretical development, a loophole-free steering experiment was reported in ref. 15, and one-way steering was demonstrated in ref. 16. In addition, steering detection based on all-versus-nothing argument was proposed in refs 17 and 18, along with an experimental demonstration^19^. Meanwhile, steering quantification has received increasing attention in the past few years^8^,^20^,^21^, which leads to several useful steering measures, such as steerable weight^20^ and steering robustness^8^.

Despite these fruitful achievements, steering detection and quantification have remained challenging tasks, and many basic questions are poorly understood. For example, no conclusive criterion is known for determining the steerability of generic two-qubit states except for Werner states^3^,^4^. Even for Bell-diagonal states, only a few partial results are known concerning their steerability, including several necessary criteria and several sufficient criteria^22^,^23^,^24^; further progresses are thus highly desirable. In addition, many results in the literature rely heavily on numerical calculation and lack intuitive pictures. Analytical results are quite rare since difficult optimization problems are often involved in solving steering problems.

In this work, we investigate the steerability of two-qubit Bell-diagonal states under projective measurements by the steering party. These states are appealing to both theoretical and experimental studies since they have a relatively simple structure and are particularly suitable for illustrating ideas and cultivating intuition. In addition, generic two-qubit states can be turned into Bell-diagonal states by invertible stochastic local operation and classical communication (SLOCC)^25^, so any progress on Bell-diagonal states may potentially help understand two-qubit states in general.

We first consider the steerability of Bell-diagonal states under the simplest nontrivial measurement setting on the steering party, that is, two projective measurements. We solve this problem completely by virtue of the connection between the steering problem and the joint-measurement problem^14^,^26^,^27^,^28^. In particular, we derive a necessary and sufficient steering criterion analytically and provide a simple geometrical interpretation. Such analytical results are valuable but quite rare in the literature on steering. Our study leads to a measure of steering, which turns out to equal the maximal violation of the Clauser-Horne-Shimony-Holt (CHSH) inequality^29^,^30^. As an implication, a Bell-diagonal state is steerable by two projective measurements iff it violates the CHSH inequality. This conclusion presents a sharp contrast with the observation that steering is necessary but usually not sufficient for Bell nonlocality^3^,^4^,^31^. On the other hand, in the special case of rank-2 Bell diagonal states, entanglement is sufficient to guarantee steering and Bell nonlocality, in line with the spirit of Gisin’s theorem^32^,^33^. The relations between our steering measure and concurrence as well as the volume of the steering ellipsoid are then clarified. Quite surprisingly, the steering measure and the volume of the steering ellipsoid seem to display opposite behaviors for states with given concurrence.

Finally, we explore the steerability of Bell-diagonal states under three projective measurements. Although such problems are generally very difficult to address, we derive a nontrivial sufficient criterion, which also has a simple geometrical interpretation. This criterion can detect the steerability of many states that are not steerable by two projective measurements. The relation between entanglement and steering in this scenario is also clarified.

## Setting up the stage.

Consider two remote parties, Alice and Bob, who share a bipartite quantum state *ρ* with reduced states *ρ*_A_ and *ρ*_B_ for the two parties, respectively. Alice can perform a collection of local measurements as characterized by a collection of positive-operator-valued measures (POVMs) 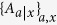, where *x* labels the POVM and *a* labels the outcome in each POVM. Recall that a POVM 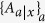 is composed of a set of positive operators that sum up to the identity, that is, 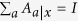. The whole collection of POVMs 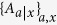 is called a *measurement assemblage*. If Alice performs the measurement *x* and obtains the outcome *a*, then Bob’s subnormalized reduced state is given by 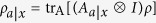. Note that 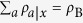 is independent of the measurement chosen by Alice, as required by the no signaling principle. The set of subnormalized states 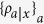 for a given measurement *x* is an *ensemble* for *ρ*_B_, and the whole collection of ensembles 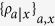 is a *state assemblage*^12^.

The state assemblage 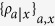 is *unsteerable* if there exists a local hidden state (LHS) model^3^,^4^,^14^,^26^,^27^,^28^:


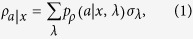


where 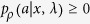, 
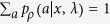
, and σ_*λ*_ are a collection of subnormalized states that sum up to *ρ*_B_ and thus form an ensemble for *ρ*_B_. This model means that Bob can interpret his conditional states 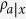 as coming from the preexisting states σ_*λ*_, where only the probabilities are changed due to the knowledge of Alice’s measurements and outcomes.

The steering problem is closely related to the joint-measurement problem. A measurement assemblage 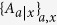 is *compatible* or *jointly measurable*^26^,^27^,^28^,^34^,^35^ if there exist a POVM 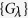 and probabilities 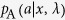 with 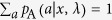 such that


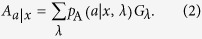


Physically, this means that all the measurements in the assemblage can be measured jointly by performing the measurement 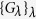 and post processing the measurement data. According to the above discussion, determining the compatibility of a measurement assemblage is mathematically equivalent to determining the unsteerability of a state assemblage. Therefore, many compatibility problems can be translated into steering problems, and vice versa^14^,^26^,^27^,^28^. This observation will play an important role in the present study.

When *ρ*_B_ is of full rank, the state assemblage 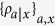 for Bob can be turned into a measurement assemblage as follows^14^,^28^,


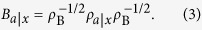


Note that the set of operators 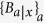 for a given *x* forms a POVM, which is referred to as Bob’s *steering-equivalent observable* (or POVM)^28^. The measurement assemblage 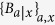 is compatible iff the state assemblage 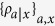 is unsteerable. For example, if 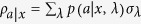, then 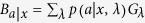 with 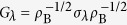; the converse follows from the same reasoning. This observation suggests a fruitful approach for understanding steering via steering-equivalent observables.

## Results

### Steer Bell-diagonal states by projective measurements

Any two-qubit state can be written in the following form


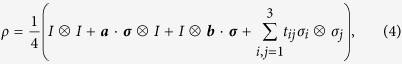


where *σ*_*j*_ for *j* = 1, 2, 3 are three Pauli matrices, ***σ*** is the vector composed of these Pauli matrices, ***a*** and ***b*** are the Bloch vectors associated with the reduced states of Alice and Bob, respectively, and 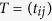 is the correlation matrix. The two-qubit state is a Bell-diagonal state iff ***a*** = ***b*** = **0 **36, in which case we have


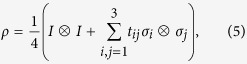


with two completely mixed marginals, that is, 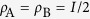. Bell-diagonal states are of special interest because they have a simple structure and are thus a good starting point for understanding states with more complex structure. In addition, all two-qubit states except for a set of measure zero can be turned into Bell-diagonal states by invertible SLOCC^25^.

With a suitable local unitary transformation, the correlation matrix *T* in (5) can be turned into diagonal form, so that


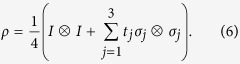


As an implication of this observation, a Bell-diagonal state is steerable by one party iff it is steerable by the other party, so there is no one-way steering^5^ for Bell-diagonal states. It does not matter which party serves as the steering party in the present study.

In the case of a qubit, any projective measurement 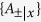 with two outcomes ± is uniquely determined by a unit vector 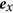 on the Bloch sphere as 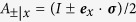. If Alice and Bob share the Bell-diagonal state (5) and Alice performs the projective measurement determined by 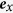, then the two outcomes will occur with the same probability of 1/2, and the subnormalized reduced states of Bob are given by 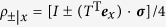. Accordingly, Bob’s steering-equivalent observable takes on the form


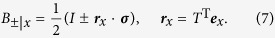


Note that this observable is uniquely characterized by the subnormalized vector 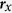, which determines an unbiased noisy (or unsharp) von Neumann observable. Here “unbiased” means that 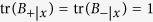. In this way, the correlation matrix *T* induces a map from projective measurements of Alice to noisy projective measurements of Bob. Alice can steer Bob’s system using the measurement assemblage 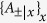 iff the set of noisy projective measurements 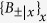 is incompatible.

**Figure 1:**
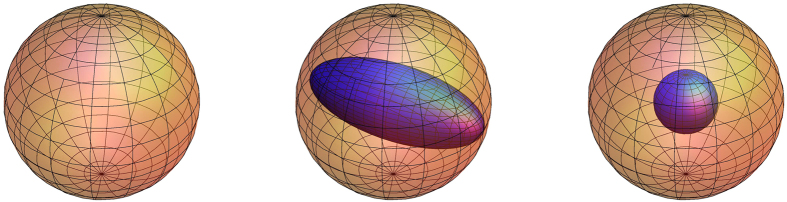
The steering ellipsoids of three Bell-diagonal states. The ellipsoid of a Bell state (left) coincides with the Bloch sphere; the ellipsoid of a rank-2 Bell-diagonal state or an edge state (middle) is rotationally symmetric with the largest semiaxis equal to the radius of the Bloch sphere; the ellipsoid of a Werner state (right) is a sphere contained in the Bloch sphere.

To see the geometric meaning of the map induced by *T*, note that the end point of 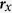 lies on an ellipsoid 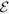 centered at origin and characterized by the symmetric matrix 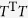: the three eigenvalues of 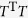 are the squares of the three semiaxes (some of which may vanish), and the eigenvectors determine the orientations of these semiaxes; see Fig. 1. This ellipsoid encodes the set of potential noisy projective measurements of Bob induced by projective measurements of Alice. Geometrically, this ellipsoid is identical to the steering ellipsoid introduced in refs 23,37 and 38, which encodes the set of states to which Alice can steer Bob’s system. It is also referred to as the steering ellipsoid here although the meaning is slightly different from that in refs 23,37 and 38. Since its discovery, the steering ellipsoid has played an important role in understanding various features pertinent to entanglement and steering^23^,^24^,^37^,^38^,^39^. To appreciate its significance in the current context, note that the steerability of a Bell-diagonal state by the measurement assemblage 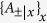 is completely determined by the set of vectors 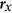 on the steering ellipsoid. Moreover, in several cases of primary interest to us, the steerability can be determined by purely geometrical means, as we shall see shortly.

### Steering by two projective measurements

In this section we derive a necessary and sufficient criterion on the steerability of a Bell-diagonal state under two projective measurements. We also introduce a steering measure and illustrate its geometrical meaning. Our study shows that a Bell-diagonal state is steerable by two projective measurements iff it violates the CHSH inequality. Furthermore, we clarify the relations between entanglement, steering, and Bell nonlocality by deriving tight inequalities between the following three measures: the concurrence, the steering measure, and the volume of the steering ellipsoid.

**Theorem 1.** *A Bell-diagonal state with correlation matrix T is steerable by two projective measurements iff* 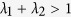, *where* 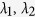 *are the two largest eigenvalues of* 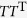.

*Proof*. Suppose Alice performs two projective measurements 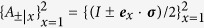. Then Bob’s steering equivalent observables are given by 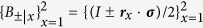, where 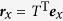, as specified in (7). According to ref. 40 (see also refs 35 and 41, 42, 43), the two observables are compatible iff



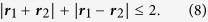



Note that 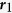 and 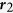 are two vectors on the steering ellipsoid, and the left hand side of the inequality is half of the perimeter of a parallelogram inscribed on the steering ellipsoid, with the plane spanned by the parallelogram passing the centre of the ellipsoid. So the Bell-diagonal state is steerable iff the maximal perimeter of such parallelograms is larger than 4. Interestingly, the maximum can be derived with a similar method used for deriving the maximal violation of the CHSH inequality^30^,^44^,


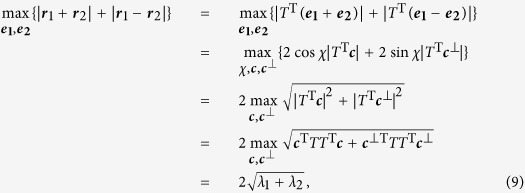


where 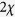 is the angle spanned by 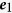 and 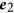; ***c*** and 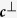 are the direction vectors of 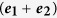 and 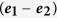, respectively, which are always orthogonal. Here the maximum in the last step is attained when ***c*** and 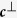 span the same space as that spanned by the two eigenvectors associated with the two largest eigenvalues of 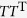. The maximum over 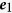 and 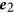 can be attained when the two vectors are eigenvectors corresponding to the two largest eigenvalues of 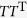. The Bell-diagonal state is steerable by two projective measurements iff 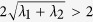, that is, 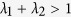. □

The choices of ***c*** and 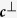 that maximize (9) are highly not unique. Therefore, the optimal projective measurements that Alice needs to perform are also not unique. Although the optimal measurements can always be chosen to be mutually unbiased as shown in the above proof, it is usually not necessary to do so. As an example, consider the Bell-diagonal state characterized by the correlation matrix 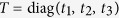 with 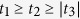. One choice of ***c*** and 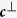 reads 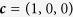 and 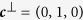, which leads to the optimal measurement directions 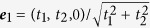 and 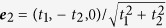. Note that 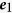 and 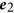 are not orthogonal in general, so the corresponding projective measurements are not mutually unbiased.

The proof of Theorem 1 also suggests a steering measure of a Bell-diagonal state under two projective measurements, namely, 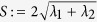. This measure has a simple geometrical meaning: 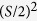 is equal to the sum of squares of the two largest semiaxes of the steering ellipsoid. A Bell-diagonal state is steerable in this scenario iff 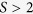. The maximum 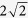 of *S* is attained when 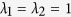, which corresponds to a Bell state. To obtain a normalized measure of steering, we may opt for 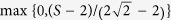. According to ref. 30, the maximal violation of the CHSH inequality by the Bell-diagonal state is equal to 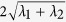 (cf. ref. 45 for a geometrical interpretation), which coincides with the steering measure *S* introduced here. This observation has an important implication for the relation between steering and Bell nonlocality.

**Corollary 1.**  *A Bell-diagonal state is steerable by two projective measurements iff it violates the CHSH inequality.*

To clarify the geometric meaning of Theorem 1 and the steering measure *S*, it is convenient to choose a concrete Bell basis. Here we shall adopt the following choice^46^,


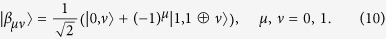


Note that 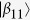 is the singlet. Thanks to the choice of the Bell basis, the correlation matrices of the four Bell states are diagonal as given by 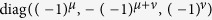. Up to a local unitary transformation, any Bell-diagonal state is a mixture of the four Bell states. Without loss of generality, we can focus on Bell-diagonal states of this form, whose correlation matrices are also diagonal, as in (6).

**Figure 2:**
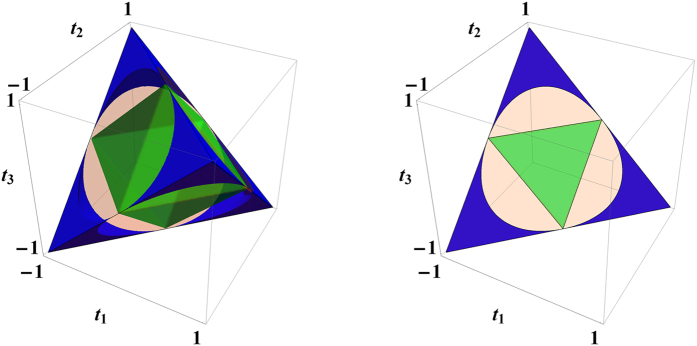
Geometric illustration of Bell-diagonal states steerable by two projective measurements. (left) The regular tetrahedron represents the set of Bell-diagonal states. The octahedron in green represents the set of separable states. The blue regions represent those states that are steerable by two projective measurements. (right) A face of the regular tetrahedron which represents the set of rank-3 Bell-diagonal states.

Geometrically, the set of Bell-diagonal states forms a regular tetrahedron, whose vertices correspond to the four Bell states^36^,^46^. The set of separable Bell-diagonal states forms an octahedron inside the tetrahedron^36^,^46^. The tetrahedron can be embedded into a cube whose sides are aligned with the three axes labelled by 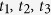, as shown in Fig. 2. In this way, a Bell-diagonal state is uniquely specified by its three coordinates 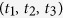. The half steering measure *S*/2 of this Bell-diagonal state is equal to the maximum over 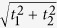, 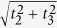, and 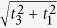, which is equal to the maximal length of the three projections of 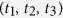 onto the three coordinate planes. Note that *S* is convex in 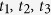 and defines a norm in the three-dimensional vector space that accommodates Bell-diagonal states. Each level surface of this norm is determined by three orthogonal cylinders of equal radius. In particular, the set of unsteerable Bell-diagonal states (determined by the level surface with 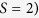 is contained in the intersection of the three solid cylinders specified by the following three inequalities, respectively,


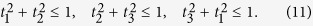


In the rest of this section we clarify the relations between the following three measures: the concurrence, the steering measure *S*, and the volume of the steering ellipsoid. Since *S* is equal to the maximal violation of the CHSH inequality, our discussion is also of interest to studying Bell nonlocality.

Recall that a two-qubit state is entangled iff it has nonzero concurrence and that the concurrence of a Bell-diagonal state is given by 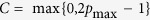, where 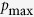 is the maximal eigenvalue of the state^47^. Given a Bell-diagonal state with correlation matrix *T*, the normalized volume *V* of the steering ellipsoid is defined as 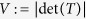
23. If *T* is diagonal, say 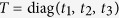, then 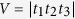. The constraints 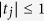 for 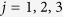 imply that 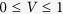, where the upper bound is saturated only for Bell states.

Calculation shows that 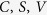 satisfy the following inequalities (see Methods section for more details):


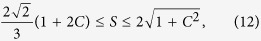



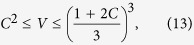



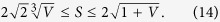


Here the lower bound in (12) is applicable to entangled Bell-diagonal states, while the other five bounds in (12), (13), and (14) are applicable to all Bell-diagonal states. The inequality 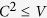 was also derived in ref. 39. As an implication of the above inequalities, any Bell-diagonal state with concurrence larger than 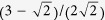 is steerable by two projective measurements. The normalized volume of the steering ellipsoid of any separable Bell-diagonal state is bounded from above by 1/27, in agreement with the result in ref. 23, while that of any unsteerable Bell-diagonal state is bounded from above by 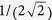.

Two types of Bell-diagonal states deserve special attention as they saturate certain inequalities in (12), (13), and (14). A Werner state has the form


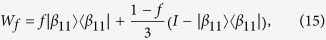


where 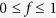. Note that *f* is equal to the singlet fraction when 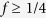. Geometrically, the Werner state lies on a diagonal of the cube in Fig. 2; conversely, any Bell-diagonal state lying on a diagonal of the cube is equivalent to a Werner state under a local unitary transformation. The correlation matrix for the Werner state has the form 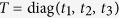 with 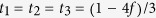. Therefore, the steering ellipsoid reduces to a sphere with radius 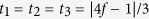; see the right plot in Fig. 1. In addition,


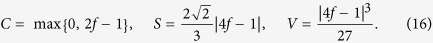


The Werner state is steerable by two projective measurements iff 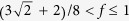. It saturates the lower bound in (14) and, when 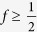, also the lower bound in (12) and the upper bound in (13).

Those states lying on an edge of the tetrahedron in Fig. 2 are called *edge states* (or rank-2 Bell-diagonal states). If an edge state has two nonzero eigenvalues *p* and 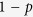 with 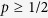, then 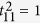 and 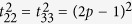 (assuming 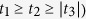. Therefore, the steering ellipsoid is rotationally symmetric with the largest semiaxis equal to 1 and the other two semiaxes equal to 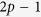; see the middle plot in Fig. 1. In addition,


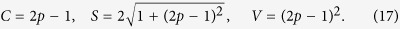


The edge state is steerable by two projective measurements whenever 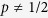, that is, when it is entangled. So entanglement is sufficient to guarantee steering and Bell nonlocality in this special case, which complements Gisin’s theorem^32^,^33^. In addition, the edge state saturates the upper bounds in (12) and (14) as well as the lower bound in (13).

**Figure 3:**
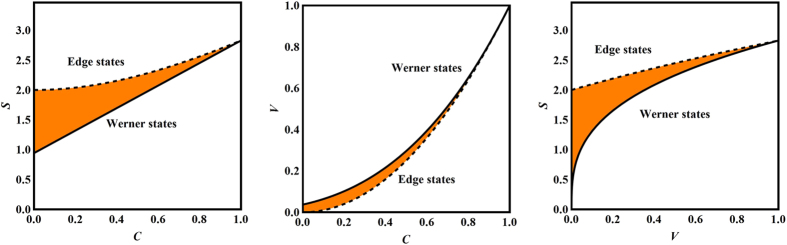
Relations between three entanglement and steering measures for Bell-diagonal states. Here *C* is the concurrence, *S* is the steering measure, and *V* is the normalized volume of the steering ellipsoid. The orange region in each plot indicates the range of values. The dashed lines represent edge states and the solid lines represent Werner states.

Figure 3 illustrates the relations between *C*, *S*, *V*. When the concurrence *C* is large, the three measures are closely correlated to each other, while they tend to be more independent in the opposite scenario. Quite surprisingly, the normalized volume *V* of the steering ellipsoid seems to have a closer relation with concurrence *C* rather than the steering measure *S*. In addition, for given concurrence *C* > 0, the volume *V* attains the maximum when the steering measure *S* attains the minimum, and vice versa.

### Steering by three projective measurements

In this section we explore the steerability of Bell-diagonal states under three projective measurements by the steering party. To this end, we need a criterion for determining the compatibility of three unbiased noisy projective measurements. Fortunately, this problem has been solved in refs 48 and 49, according to which, three noisy binary observables 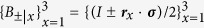 are compatible if


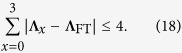


Here 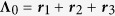, 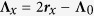 for 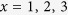, and 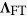 denotes the Fermat-Toricelli (FT) vector of 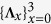, which is the vector 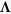 that minimizes the total distance 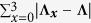. In general, 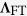 has no analytical expression^48^,^49^.

Given a Bell-diagonal state with correlation matrix *T*, suppose Alice performs three projective measurements 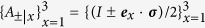. Then Bob’s steering equivalent observables are given by 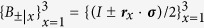, where 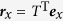 for 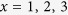. Define


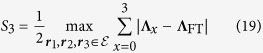


as a steering measure of the Bell-diagonal state under three projective measurements, where 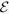 is the steering ellipsoid. Then the Bell-diagonal state is steerable by three projective measurements iff 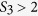. In general, it is not easy to compute 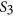. Here we shall derive a nontrivial lower bound, which is very useful for understanding the steerability of Bell-diagonal states by three projective measurements.

When 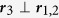, the FT vector can be determined explicitly^49^ (note that there is a typo in ref. 49 about the sign),


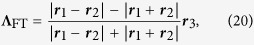


which imply that


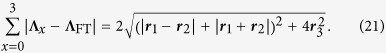


**Theorem 2.** *Any Bell-diagonal state with* 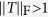 *is steerable by three projective measurements, where* 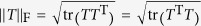 *is the Frobenius norm of the correlation matrix* 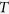.

*Proof*. Let 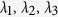 be the eigenvalues of 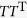 in nonincreasing order and 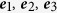 the associated orthonormal eigenvectors. Let 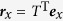 for 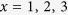. Then 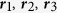 are mutually orthogonal and



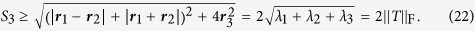



If the Bell-diagonal state is not steerable by three projective measurements, then 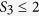, so 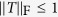. □

**Figure 4:**
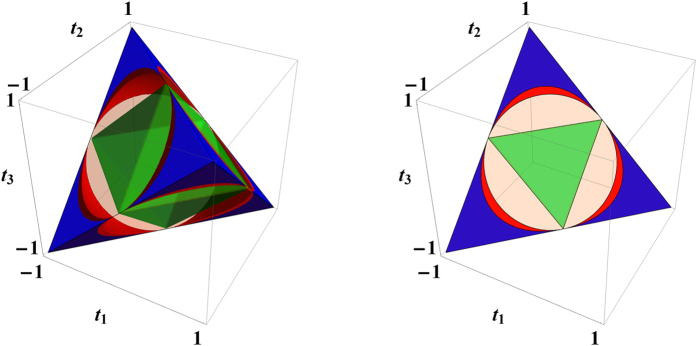
Illustration of Bell-diagonal states steerable by three projective measurements (cf. Fig. 2). (left) The regular tetrahedron represents the set of Bell-diagonal states. The octahedron in green represents the set of separable states. The blue regions represent those states that are steerable by two projective measurements, and the red regions represent those states that are not steerable by two projective measurements but steerable by three projective measurements as specified in the proof of Theorem 2. (right) A face of the regular tetrahedron which represents the set of rank-3 Bell-diagonal states.

The Frobenius norm 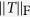 happens to be the Euclidean norm of the vector 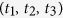 that represents the Bell-diagonal state in Figs 2 and 4; its square is equal to the sum of squares of the three semiaxes of the steering ellipsoid (cf. ref. 50). The set of Bell-diagonal states with the same norm 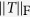 lies on a sphere. It is clear from the above discussion that 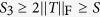, so any Bell-diagonal state that is steerable by two projective measurements is also steerable by three projective measurements, as expected. The converse is not true in general, as illustrated in Fig. 4. Consider the Werner state in (15) for example, we have 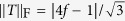, so the Werner state is steerable by three projective measurements if 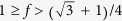. By contrast, it is steerable by two projective measurements only if 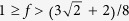.

The relations between 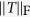 and *C*, *V* can be derived with similar methods used in deriving (12) and (14), with the results


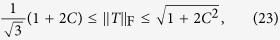



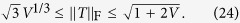


**Figure 5:**
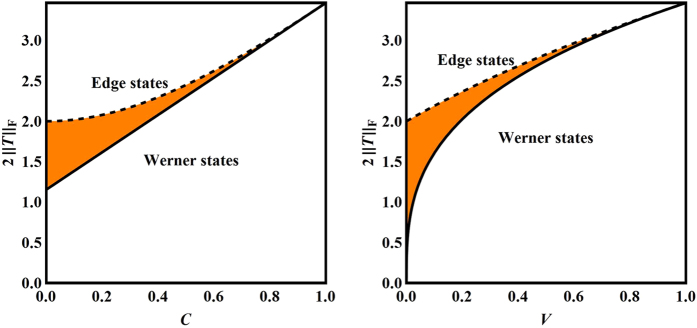
Ranges of values of 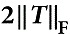 versus the concurrence *C* (left) and the normalized volume *V* of the steering ellipsoid (right) for Bell-diagonal states. Here 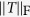 is the Frobenius norm of the correlation matrix *T*, and 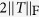 is a lower bound for the steering measure *S*_3_, which determines the steerability of Bell-diagonal states under three projective measurements. The dashed lines represent edge states and the solid lines represent Werner states.

Here the lower bound in (23) is applicable to entangled Bell-diagonal states, while the other three bounds are applicable to all Bell-diagonal states. As in (12) and (14), the two lower bounds are saturated by Werner states, while the two upper bounds are saturated by edge states; see Fig. 5. These inequalities are quite instructive to understanding the steering of Bell-diagonal states by three projective measurements given that 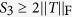. As an implication, any unsteerable Bell-diagonal state satisfies 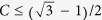 and 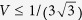.

## Discussion

In summary, we studied systematically the steerability of Bell-diagonal states by projective measurements on the steering party. In the simplest nontrivial scenario of two projective measurements, we solved the problem completely by deriving a necessary and sufficient criterion, which has a simple geometrical interpretation. We also introduced a steering measure and proved that it is equal to the maximal violation of the CHSH inequality. This conclusion implies that a Bell-diagonal state is steerable by two projective measurements iff it violates the CHSH inequality. In the special case of edge states, our study shows that entanglement is sufficient to guarantee steering and Bell nonlocality. In addition, we clarified the relations between entanglement and steering by deriving tight inequalities satisfied by the concurrence, our steering measure, and the volume of the steering ellipsoid. Finally, we explored the steerability of Bell-diagonal states under three projective measurements. A simple sufficient criterion was derived, which can detect the steerability of many states that are not steerable by two projective measurements.

Our study provided a number of instructive analytical results on steering, which are quite rare in the literature. These results not only furnish a simple geometric picture about steering of Bell-diagonal states, but also offer valuable insight on the relations between entanglement, steering, and Bell nonlocality. They may serve as a starting point for exploring more complicated steering scenarios. In addition, our work prompts several interesting questions, which deserve further studies. For example, is the steering criterion in Theorem 2 both necessary and sufficient? Is there any upper bound on the number of measurements that are sufficient to induce steering for all steerable Bell-diagonal states? We hope that these questions will stimulate further progress on the study of steering.

## Methods

### Concurrence and steering measure

Here we derive the inequalities in (12), (13), and (14) in the main text, which characterize the relations between the concurrence *C*, the steering measure *S* (under two projective measurements), and the volume *V* of the steering ellipsoid. We also determine those Bell-diagonal states that saturate these inequalities. Similar approach can be applied to derive (23) and (24), which are pertinent to steering of Bell-diagonal states by three projective measurements.

Without loss of generality, we may assume that *ρ* has the form in (6) with 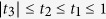. Then the spectrum of *ρ* is given by


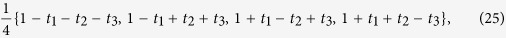


where the eigenvalues are arranged in nondecreasing order. The minimal and the maximal eigenvalues are respectively given by 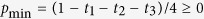 and 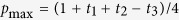.

If the Bell-diagonal state is separable, that is 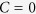, then 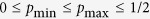 36, which implies that


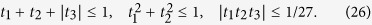


So the inequalities 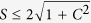, 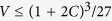, and 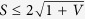 in (12), (13), and (14) hold for separable Bell-diagonal states. The inequality 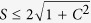 is saturated iff 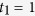, 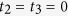, in which case *ρ* is an edge state with two nonzero eigenvalues equal to 1/2. The inequality 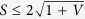 is saturated under the same condition. The inequality 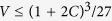 is saturated iff 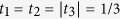, in which case *ρ* is a Werner state which either has singlet fraction 1/2 or is proportional to a projector of rank 3. Here states that are equivalent to 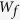 in (15) under local unitary transformations are also called Werner states. The inequality 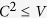 in (13) is trivial for separable states; it is saturated iff 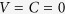, that is, 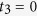, in which case the Bell-diagonal state lies on a coordinate plane in Fig. 2. The inequality 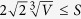 follows from the definitions of *S* and *V* and is applicable to both separable and entangled states. It is saturated iff 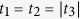, in which case *ρ* is a Werner state.

If the Bell-diagonal state is entangled, then 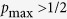, 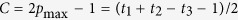, and 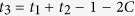. The positivity of *ρ* and the requirement 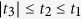 lead to the following set of inequalities,


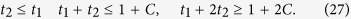


These inequalities determine a triangular region in the parameter space of 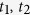 with the following three vertices:


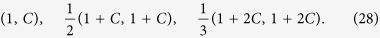


The maximum 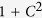 of 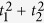 under these constraints is attained iff 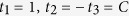, in which case the state has two nonzero eigenvalues equal to 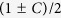 and is thus an edge state. The minimum 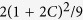 is attained iff 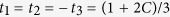, in which case the state has one eigenvalue equal to 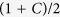 and three eigenvalues equal to 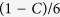, and is thus a Werner state. By contrast, the maximum 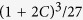 of 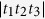 is attained exactly when 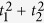 attains the minimum, and the minimum 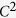 of 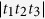 is attained when 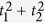 attains the maximum. Therefore, (12) and (13) hold for entangled Bell-diagonal states. As an immediate corollary, (14) also holds in this case.

In summary, the lower bound in (12) is applicable to entangled Bell-diagonal states, while the other five bounds in (12), (13), (14) are applicable to all Bell-diagonal states. The two inequalities 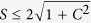 and 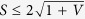 are saturated only for edge states. The inequality 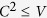 is saturated only for edge states and those states with 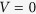. The two inequalities 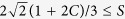 and 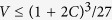 are saturated only for Werner states that have singlet fractions at least 1/2 or Werner states that are proportional to rank-3 projectors. The inequality 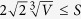 is saturated only for Werner states. In particular, among entangled Bell-diagonal states, only edge states and Werner states with singlet fractions larger than 1/2 can saturate these inequalities.

## Additional Information

**How to cite this article**: Quan, Q. *et al.* Steering Bell-diagonal states. *Sci. Rep.* **6**, 22025; doi: 10.1038/srep22025 (2016).
